# Predicting recovery following stroke: Deep learning, multimodal data and feature selection using explainable AI

**DOI:** 10.1016/j.nicl.2024.103638

**Published:** 2024-07-02

**Authors:** Adam White, Margarita Saranti, Artur d’Avila Garcez, Thomas M.H. Hope, Cathy J. Price, Howard Bowman

**Affiliations:** a Department of Computer Science, City, University of London, UK; b School of Psychology, University of Birmingham, UK; c School of Computer Science, University of Birmingham, UK; d Wellcome Centre for Human Neuroimaging, University College London, UK

## Abstract

Machine learning offers great potential for automated prediction of post-stroke symptoms and their response to rehabilitation. Major challenges for this endeavour include the very high dimensionality of neuroimaging data, the relatively small size of the datasets available for learning and interpreting the predictive features, as well as, how to effectively combine neuroimaging and tabular data (e.g. demographic information and clinical characteristics). This paper evaluates several solutions based on two strategies. The first is to use 2D images that summarise MRI scans. The second is to select key features that improve classification accuracy. Additionally, we introduce the novel approach of training a convolutional neural network (CNN) on images that combine regions-of-interests (ROIs) extracted from MRIs, with symbolic representations of tabular data.

We evaluate a series of CNN architectures (both 2D and a 3D) that are trained on different representations of MRI and tabular data, to predict whether a composite measure of post-stroke spoken picture description ability is in the aphasic or non-aphasic range. MRI and tabular data were acquired from 758 English speaking stroke survivors who participated in the PLORAS study. Each participant was assigned to one of five different groups that were matched for initial severity of symptoms, recovery time, left lesion size and the months or years post-stroke that spoken description scores were collected. Training and validation were carried out on the first four groups. The fifth (lock-box/test set) group was used to test how well model accuracy generalises to new (unseen) data.

The classification accuracy for a baseline logistic regression was 0.678 based on lesion size alone, rising to 0.757 and 0.813 when initial symptom severity and recovery time were successively added. The highest classification accuracy (0.854), area under the curve (0.899) and F1 score (0.901) were observed when 8 regions of interest were extracted from each MRI scan and combined with lesion size, initial severity and recovery time in a 2D Residual Neural Network (ResNet). This was also the best model when data were limited to the 286 participants with moderate or severe initial aphasia (with area under curve = 0.865), a group that would be considered more difficult to classify.

Our findings demonstrate how imaging and tabular data can be combined to achieve high post-stroke classification accuracy, even when the dataset is small in machine learning terms. We conclude by proposing how the current models could be improved to achieve even higher levels of accuracy using images from hospital scanners.

## Highlights

• A multimodal deep learning method is presented, which combines structural brain scans with tabular features.
• This multimodal approach can be applied to classifying stroke patients as aphasic versus non-aphasic.
• Classification accuracies in the low to mid 80% can be reached.
• Feature selection using explainable-AI can be applied to structural brain-scans.
• A combination of multimodal deep learning and a focus on key brain areas provides the highest accuracy.

## 1 Introduction

Modern healthcare has become good at keeping patients alive following a stroke. Consequently, there are increasingly many stroke-survivors with debilitating impairments that they may live with for many years. Of impairments following stroke, language deficits can be particularly distressing, since they limit the ability to communicate with others, impacting relationships with friends and family, as well as work opportunities. Accordingly, post-stroke rehabilitation is critically important.

Targeted therapy for post-stroke aphasia has been shown to bring benefit, even in the chronic stage (Menahemi-Falkov et al., 2022, Pierce, 2023). Ideally, one would like to predict deficits soon after stroke and use that information to target rehabilitation at the identified deficit. Furthermore, one would like prediction of deficits to be obtained automatically, or at least with the assistance of modern machine learning.

Modern AI, through its focus on deep learning, offers great potential for automated prediction (Roohani et al., 2018, Chauhan et al., 2019). However, although there is a determined effort to acquire large datasets in stroke research, they remain small in machine learning terms. This means that the signal-to-noise level is relatively low, and this has the consequence that feature selection is likely to be needed. For example, a high resolution T1 weighted MRI scan has hundreds of thousands of voxels (features), and the number of trainable parameters in a 3D convolutional neural network (CNN) can be in the millions. Yet the number of patients in the stroke datasets rarely exceeds the low thousands. This is very little data to train networks of such high dimensionality. This paper proposes and evaluates two possible strategies. The first is to use 2D images that summarise MRI scans. The second is to identify key symbolic features to be added to image processing that can lead to better classifications.

A further challenge is how to combine the images with the symbolic features, i.e. the MRI data with tabular data (demographic information and clinical characteristics). As we will illustrate, there has only been limited success in developing multimodal deep learning systems that combine MRI and tabular data. But, as discussed below, there is robust evidence that both MRI and tabular data have value in predicting post-stroke language deficits.

In this paper, our objectives are to:

1) provide a state-of-the-art assessment of the effectiveness of deep learning when predicting a functionally informative measure of language deficits (spoken picture description) for stroke survivors, who were assessed months or years post-stroke;
2) assess the value of multimodal deep learning models, which include both images and tabular data; and
3) determine the key “information-bearing” feature dimensions in brain-scans; i.e the most important regions-of-interest, as part of an explainable AI approach.

We introduce our novel approach for training CNNs on images that combine regions-of-interest (ROIs) extracted from MRIs, with symbolic representations of tabular data.

Experiments were carried out with a series of CNN architectures (both 2D and a 3D) that combined MRI and tabular data to predict whether spoken picture description scores were in the aphasic or non-aphasic range. Several of our experiments used a Residual Neural Network (ResNet) model, as this type of CNN has been shown to provide state of the art levels of accuracy in medical imaging, due to its “skip connections” enabling the scaling-up to large numbers of layers (Yu et al. 2021). There are a variety of 2D and 3D ResNet models, typically labelled with a number following “ResNet” (e.g. ResNet-18) that refers to the number of layers in the model.

All analyses were carried out using MRI and tabular data from the Predicting Language Outcome and Recovery After Stroke (PLORAS) database (Seghier et al., 2016). This includes patients’ high resolution T1-weighted structural MRI brain scans that are acquired months or years post stroke, lesion images derived from the MRIs, and tabular data including language and cognitive scores from the Comprehensive Aphasia Test (CAT) battery (Swinburn et al., 2004). Although, we do not predict on the full range of CAT scores, but rather subdivide that range into Healthy and Impaired and perform classification on this binary distinction, since modern deep learning techniques are focussed on classification problems. PLORAS excludes patients with evidence of other neurological conditions. To ensure that low language scores were not a consequence of non-stroke related language proficiency, we also excluded patients whose native language was not English.

Hope et al. (2013) employed Gaussian process regression models to predict the CAT spoken picture description scores that are also of interest in the current study. A baseline model using just demographic data and elapsed time since stroke gave an R-squared of 0, using data from 270 patients from the PLORAS database (Seghier et al., 2016). The R-squared was increased to 0.33 when adding lesion volume; and 0.59 when adding lesion loads that indicate the proportions of anatomically defined grey and white matter regions of interest (ROI) that are categorised as “lesioned” in each patient.

Hope et al. (2018) analysed whether disrupted white matter connectivity adds unique prognostic information for post-stroke aphasia recovery. Baseline regression models were fitted using the PLORAS data of 818 patients, including demographic data, elapsed time since stroke, lesion volume and lesion loads of grey matter ROIs, where lesion load was the proportion of each ROI damaged in each binary lesion image. The baseline models were then compared to a series of models that added or replaced the data from the baseline model with white matter connectivity data. The best Pearson R scores reported for the spoken description score were 0.73. Overall, it was found that adding connectivity data did not improve prediction accuracy for patient language skills, a finding that was also observed in an independent dataset by Zhao et al. (2023). Hope et al. emphasise that their findings do not exclude white matter disruption being a key casual mechanism for post-stroke cognitive symptoms. This is because lesions may result in highly correlated grey matter and white matter damage. Hence grey matter damage could be a suitable proxy in prognostic models, even if white matter damage is etiologically important.

**Fig. 1:**
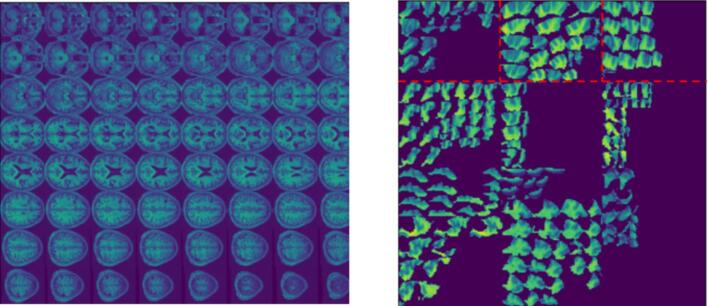
Left: An example of a stitched MRI consisting of sixty-four axial cross-sectional slices from an MRI scan. Right: An ROI Image consisting of the 12 key (most predictive) ROIs (see Section 3.2). The dotted red lines have been added to this figure for visual clarity, demarcating the boundaries of the left superior temporal gyrus, middle temporal gyrus and inferior frontal gyrus-triangular. (For interpretation of the references to colour in this figure legend, the reader is referred to the web version of this article.)

Roohani et al. (2018) trained a CNN using 2-D stitched images created from 1,211 PLORAS MRI scans. Each image consisted of sixty-four axial cross-sectional slices from each MRI scan (Fig. 1, left). The slices were always stitched in the same order, so that a voxel location in the stitched images always corresponded to the same brain location. Roohani et al. motivated their stitched image format on the grounds that there was insufficient data to effectively train a 3D network. By contrast, using 2D stitched images reduces the number of trainable parameters, whilst still capturing contextual information across scans. The CNN achieved a prediction accuracy of 79 % at classifying patients’ spoken picture description scores (aphasic or not aphasic), based on a threshold score of 60 on spoken picture description. A second analysis was carried out by combining the feature vector from the final convolutional layer with demographic data, and then regressing against spoken description scores, giving an R-squared of 0.6. Roohani et al.’s analysis suggests that the stitched image format successfully captures the predictive signal within an MRI scan, however it is not directly comparable with either of the Hope et al., 2013, Hope et al., 2018 papers, as each uses a different subset of participants from the PLORAS database.

Chauhan et al. (2019) compared the performance of a 3D CNN trained on post-stroke MRI scans with both a ridge regression and a support vector regression model trained on features of lesion images extracted by principal component analysis. A hybrid model was also trained that combined the lesion image features with features extracted from the 3D CNN. This was carried out with data from 98 patients with language deficits from a Washington University School of Medicine dataset. The support vector regression had the highest R-squared of 0.66 compared to the 3D CNN’s 0.63.

There are very few published multimodal CNNs that combine MRI data with tabular clinical data. We are unaware of any papers using multimodal CNNs for predicting language outcomes after stroke, however there are several papers on diagnosing Alzheimer’s that are relevant. Esmaeilzadeh et al. (2018) and Liu et al. (2018) both use ‘Early Fusion’ models (Huang et al., 2020). Early Fusion models consist of a CNN that learns a latent representation of the input images. The latent representation is then concatenated with the tabular data, before being passed through some fully connected layers. The Liu et al. (2018) model first identifies discriminative anatomical landmarks from MRI images, extracts image patches around these landmarks and passes these patches to a CNN. The feature maps from the last convolutional layer are then concatenated with demographic data before being passed through some fully connected layers. Wolf et al. (2022) criticise such approaches as failing to enable fine grained interaction between voxels and tabular data. They propose a multimodal 3D convolution neural network called Dynamic Affine Feature Map Transform (DAFT). DAFT employs a modified 3D ResNet architecture in which tabular data scales and shifts the feature maps of the ResNet’s final layer. Wolf et al. trained DAFT on MRI and tabular data for diagnosing and predicting Alzheimer’s disease. In their experiments, DAFT had higher balanced accuracy, AUC and F1 scores than either a baseline linear regression, an Early Fusion model, or a 3D ResNet.

The remainder of the paper is organised as follows. Section 2 specifies the data that was extracted from the PLORAS dataset, and how this was used to create new image datasets displaying features such as ROIs and symbolic representations of tabular data. A summary is also provided of an explainable AI method called CLEAR Image that was used to identify key ROIs. Section 3 specifies the experiments that were carried out using a variety of CNN architectures. It also explains how the project countered the danger of overfitting by employing a strategy of cross-validation with a hold out ‘lock box’ (Hosseini et al., 2020). The results are presented in Section 4, highlighting the potential of using images that combine ROIs and symbolic representations of tabular data. Section 5 discusses our findings and indicates directions for future work. Section 6 identifies key limitations, including that the MRI data was restricted to research quality scanners. Section 7 concludes the paper.

## 2 Methods and materials

### 2.1 Dataset

The participants were 758 S survivors from the PLORAS database (Seghier et al., 2016). The male to female ratio was 2.3:1, and the average age at stroke was 56.1. Patients included in the study could have bilaterial, left sided or right sided strokes. The dataset used for the current study consists of MRI scans, their associated tabular data and two-dimensional image datasets that are derived from the MRI scans (see 2.1.1, 2.1.2, 2.1.3, 2.1.4). Three PLORAS tabular features were identified *a priori* as being of prognostic relevance to recovery from aphasia: (i) Initial severity of aphasia after stroke (henceforth: initial severity), see Lazar et al., 2010, Benghanem et al., 2019; (ii) Left hemisphere lesion size (henceforth: left lesion size), see Hope et al., 2013, Thye and Mirman, 2018, Benghanem et al., 2019; (iii) Recovery time − which is defined as the time between the stroke and the CAT tests, see Hope et al., 2013, Johnson et al., 2022.

In this paper, initial severity was assessed by patient report (as in Roberts et al., 2022). A patient was classified as *severe* if they were conscious, physically capable of attempting to speak, but unable to speak due to aphasia; *moderate* if they were able to produce words, but not sentences; *mild* if they could produce lexically meaningful short sentences and *normal* if they did not report an impairment. There is an additional category for patients who were either unconscious and hence could not be tested, or whose score was missing. Initial severity was treated as a categorical rather than ordinal measure because the unconscious/missing values cannot be ranked relative to the other values. Initial severity scores were distributed: 25.5 % severe, 12.3 % moderate, 24.8 % mild, 17.2 % normal and 20.2 % unconscious or missing.

The outcome of interest for this paper was the total score from the CAT spoken picture description task, to be classified as either *Healthy* or *Impaired*. This task requires participants to conceptualise events in a scene, retrieve the words associated with the objects and actions, formulate sentences, and generate the associated speech sounds. It objectively measures the building blocks of connected speech, including the number and appropriateness of information carrying words, syntactic variety, speed ratings and grammatical accuracy. We focused on predicting the overall score (strictly performing a binary classification on it), which provides a reasonable proxy for participants' language skills in more naturalistic contexts. The overall scores were standardised into T-scores (not to be confused with the t-statistic) that measure patient performance relative to an independent sample of participants without aphasia. That is, the T-scores are defined relative to a separate distribution of scores on the same task, acquired from a sample of 27 neurologically normal controls. The T-scores are preferred to raw scores because they more directly represent the extremity of impairment in these tasks. For example, a reduction of 1 raw score point corresponds to a much greater change in T-score when the raw score is already low, than when it is relatively high.

We classified scores that were less than 60 as aphasic, as this is rarely observed in participants from the PLORAS database who do not have any identifiable brain damage. That is, the PLORAS database includes clinically diagnosed strokes that may have had minimum damage: patients with no detectible damage who claim that they never experienced any speech production impairments can have scores of 60 on the CAT. The distribution of spoken picture description scores was skewed, with 34 % having a score less than 60 (i.e. in the aphasic range). For patients with severe or moderate initial severity scores, 44.5 % had spoken description scores less than 60.

The MRI scans, from our 758 participants were acquired by research-dedicated MRI scanners between 30th June 2010 and 14th March 2020 (when data collection was stopped by Covid-19 restrictions). Participants recruited prior to these dates were not included because initial severity scores were not routinely collected. Imaging data were collected using either a 1.5 T Avanto scanner, a Siemens 3 T Trio scanner or a Siemens 3 T Allegra scanner. For anatomical images acquired on the 1.5 T Avanto scanner, a 3D magnetization-prepared rapid acquisition gradient-echo (MPRAGE) sequence was used to acquire 176 sagittal slices with a matrix size of 256 × 224, yielding a final spatial resolution of 1  mm isotropic voxels (repetition time/echo time/inversion time = 2730/3.57/1000  ms). For anatomical images acquired on the other 3 T scanners, an optimised 3D modified driven equilibrium Fourier transform (MDEFT) sequence was used to acquire 176 sagittal slices with a matrix size of 256 × 224, yielding a final spatial resolution of 1  mm isotropic voxels: repetition time/echo time/inversion time = 12.24/3.56/530  ms and 7.92/2.48/910  ms at 1.5 T and 3 T, respectively (Deichmann et al., 2004). Preprocessed with Statistical Parametric Mapping software (Penny et al, 2011), these images were spatially normalized into Montreal Neurological Institute (MNI) space using a unified segmentation algorithm (Ashburner and Friston, 2005, Crinion et al., 2007) optimized for use in patients with focal brain lesions via the addition of an extra ‘lesioned-tissue’ class (Seghier et al., 2008). That is, lesion segmentation used the automated lesion identification (ALI) approach, specified by Seghier et al. (2008), which outputs a 3D whole brain binary lesion image (lesioned or not lesioned) for each patient. Left lesion size was an estimate of the number of damaged voxels in each patient’s left hemisphere binary lesion image (see Hope et al., 2013).

The dataset was partitioned into five groups, such that each group was balanced in terms of recovery time, initial severity, left lesion size and spoken description score. All training and validation was carried out on the first four groups, with the fifth group being held out as a lock box/test set. In other words, a lock box is a subset of the dataset removed from the analysis pipeline before any optimisation begins, and not accessed until after all hyperparameter adjustments and training is completed. As long as no decisions concerning the set-up or training of data is made on the lock box, which would be the case if accuracy on the lock box is only assessed once, performance on the lock box is a fair test of generalization (Hosseini et al., 2020). Thus, importantly, in this work, we have not performed a nested cross validation, in which, effectively, multiple lock-boxes are used. Our approach here could be characterised as a “single-lock-box” approach.

This variant of cross-validation also enables us to make a valid comparison between models with different levels of complexity, e.g. between logistic regression and a ResNet. The problem of complexity arises because more complex models can extract pattern from the noise in the data better than less complex ones, leading to over-fitting. Our validation set stops training approximately when over-fitting starts, and this point will be reached at different points for more or less complex models. Then, we perform an out-of-sample test using our lock-box test set. If there has been overfitting due to greater model flexibility, this “clean” test of generalisation is expected to produce a lower accuracy score, thereby penalising the more complex model. We do not include any drop-out in our neural networks, or other forms of regularisation.

#### 2.1.1 Stitched MRI dataset

The 2D stitched MRI used in this paper were produced to the same specification as used by Roohani et al. (2018). These images do not rely on any lesion segmentation processing. They are created by displaying sixty-four axial cross-sectional spatially normalised MRI slices in a single 2D 632 × 760 image (see Fig. 1, left). These 2D images are then down-sampled to 256 × 256 as part of preprocessing for the CNNs. The down-sampling leads to some distortion in the shapes of the MRI slices and also some loss of information. The degree to which the resulting images can still be used to generate accurate forecasts was one of the questions for the experiments performed in this paper.

#### 2.1.2 Regions of interest (ROI) dataset

The original 2D stitched MRI images (prior to down-sampling) were parcellated into grey and white matter anatomical ROIs. The grey-matter ROIs (from now on simply ROIs) were defined by the Automatic Anatomical Labelling atlas (Tzourio-Mazoyer et al., 2002). Those that contributed most to the ResNet-18′s predictions (see below) were considered “key anatomical ROIs” and stitched together into “ROI images” (Fig. 1, right). A possible advantage of using ROI images is that the ROIs can be kept at the original resolution of the MRI scan and hence no information is lost, whereas the stitched MRI were down-sampled (as described above). Using ROI images may also reduce the risk of the curse of dimensionality (Altman and Krzywinski, 2018) compared to the stitched MRI dataset. There are also *a priori* grounds for believing that the 2D ROI images reduce redundant dimensions. For example, the most relevant ROIs for aphasia are known to be in the left hemisphere. Furthermore, there can be a significant degree of duplication in the predictive information contained within an MRI slice, as a lesion that causes aphasia is likely to damage multiple ROIs, including some that are functionally irrelevant to aphasia (Seghier & Price, 2023).

The key ROIs were identified by first training a ResNet-18 neural network on the original stitched MRI dataset to predict spoken description scores greater or equal to 60 (i.e. full recovery). An explainable AI method called CLEAR Image (see subsection 2.2) then identified which of the 116 ROIs were most important to the ResNet-18′s predictions. CLEAR Image analysed 100 predictions made by the ResNet-18 and calculated each ROI’s average feature importance score. The key ROIs (all left hemisphere) were, in order of importance: (i) superior temporal gyrus, (ii) middle temporal gyrus, (iii) inferior frontal gyrus − triangular, (iv) postcentral gyrus, (v) supramarginal gyrus, (vi) inferior frontal gyrus − opercular, (vii) insula gyrus, (viii) caudate gyrus, (ix) temporal pole, (x) inferior parietal, (xi) middle frontal gyrus, and (xii) hippocampus gyrus. Four of these are temporal lobe regions (i, ii, ix, xii), three are parietal lobe regions (v, x, iv), and three are front lobe regions (iii, vi, xi).

Cross-validation was then used to determine the number of ROIs to include in the ROI images.

**Fig. 2:**
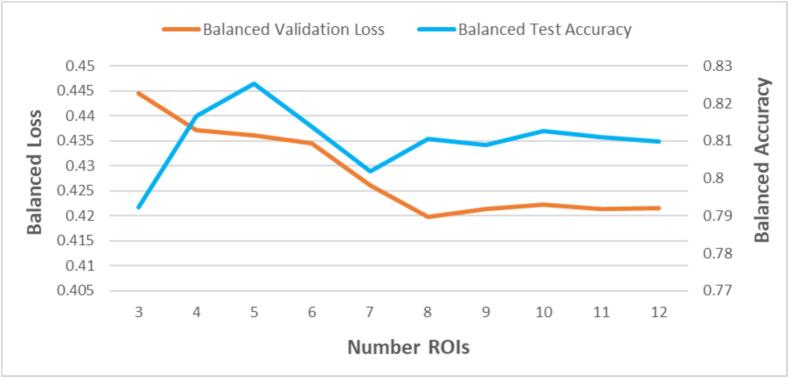
Plots of how balanced validation loss and balanced test accuracy vary with the number of ROIs displayed in ROI Images. The balanced validation loss was used to determine that 8 ROIs should be included in each ROI Image. Note that the balanced test accuracy only varies slightly with number of ROIs, achieving > 0.79 with only three ROIs.

With three ROIs, the final images would display the three highest scoring ROIs according to CLEAR Image, i.e. left superior temporal gyrus, middle temporal gyrus, inferior frontal gyrus-triangular. To determine the number of ROIs to include in the images, we used cross-validation on the original stitched MRI dataset over all combinations of the three learning rates (see below) and number of ROIs to include, which ranged from 3 to 12. It was found that the top eight ROIs minimised ResNet-18′s loss (see Fig. 2). Notice that selecting the number of ROIs based on test (i.e. lock-box) accuracy would overfit. The use of only one level of cross-validation to fit hyper-parameters, such as number of ROIs, could also induce over-fitting; however, our use of a lock box (which is only tested on after all fitting is complete) allows us to test whether our ultimate quantification of overall accuracy generalises well to new data, at least with the variability inherent to the PLORAS dataset (Hosseini et al., 2020).

#### 2.1.3 Hybrid stitched MRI dataset

**Fig. 3:**
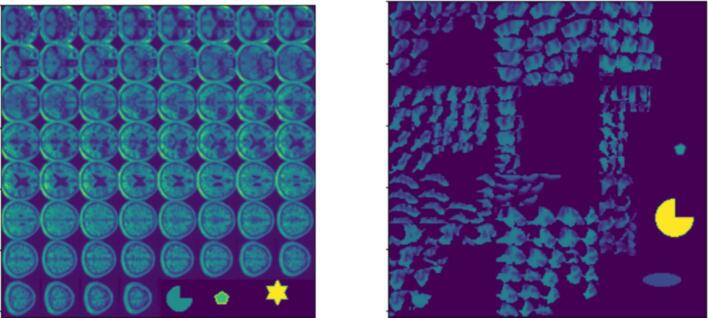
Left: A Hybrid stitched MRI, after pre-processing which reshapes it to 256 x 256. Right: A hybrid ROI image consisting of twelve ROIs plus the symbols for initial severity (normal for this patient), left lesion size and recovery time.

Hybrid stitched MRIs combine the stitched MRIs with symbolic representations of initial severity, lesion size and recovery time. The choice of symbols and how to represent feature values was largely arbitrary, the only criteria being that the neural networks to be trained following the addition of the symbols should be sensitive to these representations. Left lesion size is a continuous feature and was represented by a pentagon symbol whose radius varies in proportion to its value. Recovery time was represented by a pie-slice of fixed size whose intensity varies in proportion to its value. Each initial severity category was represented by a different symbol, for example moderate by a triangle, normal by an ellipse, and unconscious /missing by a star. In order to create space for the tabular features in the hybrid stitched MRI dataset, four MRI slices were removed (see Fig. 3., left), the excluded slices being the four most dorsal, which are rarely lesioned in our dataset.

#### 2.1.4 Hybrid ROI dataset

Hybrid ROI images combine the ROIs and the three tabular features (see Fig. 3, right). The number of ROIs displayed in each image was determined using the cross-validation process described above for the ROI dataset, but with the images now also including the three tabular features. This identified that the seven top ROIs should be included.

### 2.2 CLEAR image explainable AI system

**Fig. 4:**
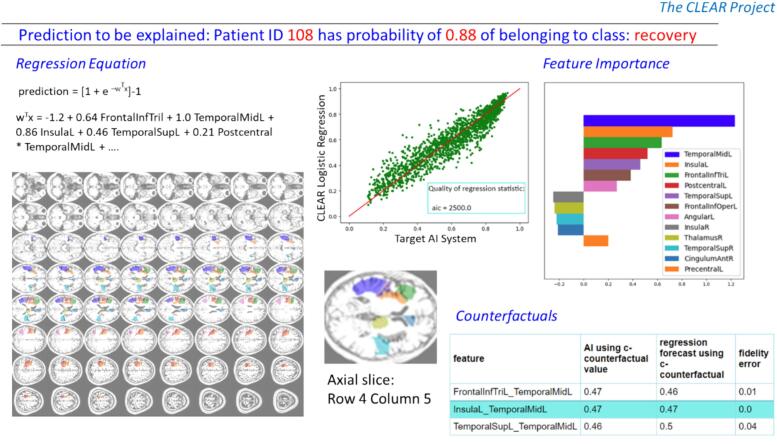
Example of a Clear Image output. This explains the classification probability determined by ResNet-18 for the stitched MRI of patient 108. CLEAR Image estimates the feature importance scores that the ResNet-18 has used in determining the classification probability. CLEAR Image also shows the logistic regression equation it generated for this stitched MRI (top left), some counterfactuals and fidelity errors – these are explained in White et al. (2023).

CLEAR Image (White et al., 2023) was used to identify which of the 116 ROIs were most salient to an image’s classification probability. CLEAR Image is a perturbation-based explainable AI method that was enhanced for this paper to use brain atlases, and also contrast MRI images. The key idea behind perturbation methods is to parcellate an image into ROIs, perturb the image, and then determine how much each ROI affects a neural network’s classification probability. Consider an example where a neural network has assigned a stitched MRI, *S,* a classification probability of 0.96. CLEAR Image creates a perturbed image *S’* by replacing an ROI of image *S* with the same ROI taken from a ‘contrast’ image *S’’* selected from a stitched MRI with a low predicted classification probability. CLEAR Image then passes the perturbed image *S*’ through the neural network and records how much the classification probability changes. By creating a large number (>1000) of perturbed images in which different combinations of ROIs are replaced and the changes in classification probability are recorded, CLEAR Image creates a regression dataset. A logistic regression is then performed, whose coefficients give the feature importance score for each ROI. An example of a CLEAR Image explanation is shown in Fig. 4. For a full specification of the CLEAR Image method and a comparison with other perturbation methods see White et al., 2023, White and Garcez, 2021.

## 3 Experiments and inference

Ten sets of experiments were initially carried out:

1. A baseline logistic regression.
2. ResNet-18 fine-tuned on the Stitched MRI dataset.
3. A lightweight CNN, based on the 2D CNN used by Roohani et al. (2018), trained on the Stitched MRI dataset.
4. 3D ResNet10 fine-tuned on the MRI scans dataset.
5. Early fusion model trained with the Stitched MRI dataset and tabular data.
6. Dynamic Affine Feature Transform (DAFT) – a multimodal 3D CNN trained on the MRI scans dataset and tabular data.
7. ResNet-18 fine-tuned on the ROI dataset.
8. ResNet-18 fine-tuned on the Hybrid Stitched MRI dataset.
9. A lightweight CNN trained on the Hybrid Stitched MRI dataset.
10. ResNet-18 fine-tuned on the Hybrid ROI dataset.

Cross-validation was used to train each of the neural networks, with the held out test dataset (i.e. the lock box) being used to determine (final) test accuracy. That is, one of our five splits was reserved as the lock-box test set, while four fold cross validation was performed on the remaining four splits, with, on each fold, three of the four providing the training set and the remaining split providing the validation set. The neural networks were trained for a maximum of 200 epochs using an early stopping rule that selected the epoch with the minimum class-weighted binary cross-entropy validation loss. This process was repeated for three learning rates: 1e-4, 5e-4, 1e-5. The neural networks were trained using stochastic gradient descent, with the exception of the lightweight neural network, which (following Roohani et al., 2018) used Root Mean Square Propagation. All neural networks used a single parameter variant of Platt scaling to calibrate the classification probabilities (Guo et al., 2017). The ResNet-18 models used pretrained weights based on the ImageNet dataset, which were fine-tuned as part of the cross validations. Each experiment was repeated for twenty random number seeds.

The evaluation metrics were unbalanced accuracy of the predictions, balanced accuracy of predictions (i.e. the average of sensitivity and specificity), area under the ROC Curve and F1-score (i.e. the harmonic mean of precision and sensitivity). These four metrics are also reported for patients with initial severity scores that are severe or moderate, as these were taken as being clinically the most difficult to predict; see (Bowman et al., 2021, Hope et al., 2019, Bonkhoff et al., 2020) for a discussion of why patients at ceiling can inflate estimates of recovery performance. The relative importance of balanced and unbalanced accuracies was a question of interest. Balanced accuracy is perhaps the easiest to interpret. However, if the proportion of patients with and without spoken-picture description deficits in our dataset reflects the presentation of patients at hospital stroke units, then unbalanced accuracy would be more representative of the effectiveness of our machine learning classification, since it would reflect the prior probability of patients presenting with particular conditions. Accordingly, unbalanced accuracy might also be a relevant measure to consider.

Additional information on some of the experiments is provided in 3.1, 3.2, 3.3, 3.4, 3.5 and then the statistical procedure we have performed is discussed in subsection 3.6.

### 3.1 Baseline regression

A binary logistic regression model was created to provide a baseline forecast for the paper. Its independent variables were the three *a priori* features: left lesion size, initial severity, recovery time. Cross-validation was not used for the logistic regression, allowing all four groups to be used for training (but excluding the fifth group, i.e. the lock box). (Although, a different approach was taken in appendix A2.).

### 3.2 Lightweight neural network trained on stitched MRI

This is the neural network used by Roohani et al. (2018). The basic building block was the commonly used sequence of a 2D convolution, followed by a ReLU function and a max pooling function. This was repeated six times. Such shallow architectures have been suggested to perform similarly to deeper networks such as ResNet-50 and Inception v-3 when applied to medical images (Raghu et al., 2019).

### 3.3 3D ResNet10 trained on MRI scans

MED3D’s ResNet10 and ResNet-18 were evaluated. These were pretrained using the 3DSeg-8 dataset, which was aggregated from several medical challenges (Chen et al., 2019).

### 3.4 Multimodal trained on both stitched MRI slices and tabular data

An early fusion model using a ResNet-18 with the Stitched MRI dataset. The feature maps from the last convolutional layer of a ResNet-18 are concatenated with the three *a priori* tabular features and then passed through a fully connected layer.

### 3.5 Dynamic Affine feature Transform (DAFT)

We use the same DAFT-Resnet model that Wolf et al. applied in their Alzheimer’s study. Their modified ResNet is lightweight, with its four blocks having 4, 8, 16 and 32 output channels respectively.

### 3.6 Comparing models

We will compare the accuracy performance of our different models. To do this, we will perform *t*-test comparisons to assess statistical robustness of the accuracy differences we observe. However, statistical inference to demonstrate that one machine learning algorithm is superior to another has its challenges (Dietterich, 1998), a major issue being, if a test is performed across folds, samples will not be independent. We discuss this issue in Appendix A1 and highlight a degrees of freedom adjustment that compensates for this loss of independence.

## 4 Results

Accuracy results are shown in Table 1. All the models performed well with balanced accuracies (all patients) exceeding 0.800. For the logistic regression (without stitched or ROI images), all three tabular features were statistically significant (p < 0.001) although, these significant findings are, to some extent, carried by the very high degrees of freedom associated with these tests (Lorca-Puls et al., 2018). If the logistic regression was run just with ‘left lesion size’ as the independent variable, the balanced accuracies dropped to 0.678/0.694 (all patients/patients with initial severity severe and moderate). Adding ‘initial severity’ increased the balanced accuracies to 0.757/0.706; further adding ‘recovery time’ gave balanced accuracies of 0.813/0.780. The logistic regression results do not have confidence intervals, as the regressions used Statsmodel’s deterministic Broyden–Fletcher–Goldfarb–Shanno optimization method (https://www.statsmodels.org/stable/optimization.html) and hence the results did not vary across folds or with random seed.

**Table 1:** Accuracy results on the lock box test data. Accuracies and confidence intervals are calculated across the four folds of our cross validation. (I = uses image data, T = uses tabular data.).

			All Patients		Initial Severity: Severe or Moderate	
	I	T	Accuracy	Balanced accuracy	Accuracy	Balanced accuracy
Logistic regression	X	Y	0.847	0.813	0.782	0.780
Stitched MRI w/ResNet-18	Y	X	0.823 ± 0.04	0.807 ± 0.03	0.746 ± 0.07	0.746 ± 0.07
Stitched MRI w/Lightweight CNN	Y	X	0.825 ± 0.04	0.801 ± 0.02	0.739 ± 0.04	0.739 ± 0.04
MRI Scans w/ResNet3D	Y	X	0.818 ± 0.02	0.805 ± 0.05	0.747 ± 0.13	0.747 ± 0.13
Early Fusion Hybrid w/ResNet-18	Y	Y	0.820 ± 0.03	0.800 ± 0.05	0.732 ± 0.09	0.732 ± 0.09
Dynamic Affine Feature Transform	Y	Y	0.818 ± 0.05	0.814 ± 0.03	0.758 ± 0.07	0.759 ± 0.07
Hybrid ROIs w/ResNet-18	Y	Y	**0.866 ± 0.02**	**0.854 ± 0.01**	**0.820 ± 0.04**	**0.821 ± 0.04**
Hybrid Stitched MRI w/ResNet-18	Y	Y	0.838 ± 0.03	0.829 ± 0.04	0.771 ± 0.08	0.771 ± 0.08
Hybrid Stitched MRI w/Lightweight CNN	Y	Y	0.829 ± 0.05	0.819 ± 0.02	0.762 ± 0.04	0.763 ± 0.05
ROIs w/ResNet-18	Y	X	0.832 ± 0.03	0.811 ± 0.05	0.764 ± 0.09	0.763 ± 0.09

The best results came from using the ResNet-18 with the Hybrid ROI images, improving balanced accuracy by approximately 0.04 compared to the baseline logistic regression. This difference was also statistically significant (false discovery rate corrected for multiple comparisons); see appendix A2. The models trained with the Stitched MRI dataset performed similarly to the ResNet3D, suggesting that the 2D images retained the key prognostic information contained in the 3D scans. The unbalanced accuracy and balanced accuracy results for severe/moderate initial severity are almost identical due to the test dataset being approximately balanced for these two groups.

The area under the ROC curve (AUC) and F1 scores are shown in Table 2. As seen in the balanced and unbalanced accuracies (Table 1), the Hybrid ROI model had the highest AUC and F1 scores. The second highest AUC score was observed for the ResNet3D (with 3D MRI Scans), which contrasts with its relatively poor performance on the accuracy metrics. Table 3 shows the comparison of unbalanced accuracies for different cutoff thresholds and confirms that the Hybrid ROI model dominated the ResNet3D at all thresholds.

**Table 2:** Area under the ROC curve and F1-scores for the test dataset. Accuracies and confidence intervals are calculated across the four folds of our cross validation. (I = uses image data, T = uses tabular data).

			All Patients		Initial Severity: Severe or Moderate	
	I	T	AUC	F1	AUC	F1
Logistic regression	X	Y	0.872	0.890	0.837	0.806
Stitched MRI w/ResNet-18	Y	X	0.873 ± 0.02	0.868 ± 0.03	0.820 ± 0.06	0.751 ± 0.08
Stitched MRI w/Lightweight CNN	Y	X	0.862 ± 0.02	0.872 ± 0.05	0.811 ± 0.02	0.749 ± 0.07
MRI Scans w/ResNet3D	Y	X	0.884 ± 0.07	0.867 ± 0.04	0.840 ± 0.15	0.762 ± 0.15
Early Fusion Hybrid w/ResNet-18	Y	Y	0.868 ± 0.01	0.867 ± 0.03	0.820 ± 0.06	0.741 ± 0.08
Dynamic Affine Feature Transform	Y	Y	0.879 ± 0.03	0.861 ± 0.05	0.833 ± 0.08	0.749 ± 0.05
Hybrid ROIs w/ResNet-18	Y	Y	**0.899 ± 0.01**	**0.901 ± 0.01**	**0.865 ± 0.03**	**0.820 ± 0.04**
Hybrid Stitched MRI w/ResNet-18	Y	Y	0.887 ± 0.02	0.879 ± 0.02	0.841 ± 0.06	0.768 ± 0.07
Hybrid Stitched MRI w/Lightweight CNN	Y	Y	0.879 ± 0.005	0.872 ± 0.05	0.831 ± 0.03	0.759 ± 0.05
ROIs w/ResNet-18	Y	X	0.877 ± 0.004	0.877 ± 0.02	0.847 ± 0.01	0.777 ± 0.06

**Table 3:** Comparison of unbalanced accuracy for different cutoff thresholds (confidence intervals are not shown for ease of reading). Accuracies are calculated across the four folds of our cross validation.

Threshold	0.1	0.2	0.3	0.4	0.5	0.6	0.7	0.8	0.9
Hybrid ROI	0.818	0.846	0.855	0.863	0.866	0.858	0.827	0.766	0.604
ResNet3D	0.808	0.825	0.828	0.827	0.818	0.799	0.749	0.658	0.497

Some additional analyses were carried out with the Hybrid ROI model, in order to understand its relatively strong performance. First, test runs were conducted to assess the contributions of its individual tabular features. New image datasets were created displaying the seven ROIs plus either one or two of the tabular features. As shown in Table 4, ‘initial severity’ was found to have the largest impact, whilst ‘left lesion size’ had a negligible or negative impact. Hybrid RM-ROI images that only included ‘initial severity’ and ‘recovery time’ achieved highest accuracies; however, this may be the result of overfitting, as the choice of features was not selected using cross-validation. The apparent negative impact of ‘left lesion size’ when included with the other two features may be due to its signal already being present in the other features (ROIs, ‘initial severity’ and ‘recovery time’), and that adding ‘left lesion size’ added noise that impaired classification. The effect of varying the depth of the ResNet architecture was also tested and it was found that increasing the depth slightly reduced the accuracies (see Table 5).

**Table 4:** Accuracy results for modified versions of the Hybrid ROI images. For example, ‘Initial severity & Left lesion size’ refers to experiments carried out with a dataset of images each displaying seven ROIs plus the symbols representing the initial severity and left lesion size features, but without recovery time. Accuracies and confidence intervals are calculated across the four folds of our cross validation.

	All Patients		Initial Severity: Severe or Moderate	
	Accuracy	Balanced accuracy	Accuracy	Balanced accuracy
No features added	0.832 ± 0.03	0.811 ± 0.05	0.764 ± 0.09	0.763 ± 0.09
Initial severity	0.851 ± 0.04	0.841 ± 0.04	0.799 ± 0.07	0.799 ± 0.07
Left lesion size	0.837 ± 0.04	0.819 ± 0.04	0.771 ± 0.06	0.771 ± 0.06
Recovery time	0.844 ± 0.02	0.826 ± 0.03	0.775 ± 0.06	0.774 ± 0.06
Recovery time & Left lesion size	0.845 ± 0.02	0.829 ± 0.02	0.785 ± 0.03	0.785 ± 0.03
Initial severity & Left lesion size	0.860 ± 0.03	0.844 ± 0.04	0.803 ± 0.07	0.803 ± 0.07
Initial severity & Recovery time	**0.872 ± 0.02**	**0.866 ± 0.03**	**0.825 ± 0.05**	**0.826 ± 0.05**
All three features added	0.866 ± 0.02	0.855 ± 0.03	0.822 ± 0.06	0.822 ± 0.06

**Table 5:** Accuracy results for ResNet models of different depths, trained on Hybrid ROI dataset. The number at the end of ResNet is the number of layers in the network. These are the four smallest Pytorch ResNet models for which ImageNet weights are available. Accuracies and confidence intervals are calculated across the four folds of our cross validation.

	ResNet-18	ResNet34	ResNet50	ResNet101
Accuracy	**0.866 ± 0.02**	0.855 ± 0.02	0.859 ± 0.03	0.857 ± 0.03
Balanced Accuracy	**0.854 ± 0.01**	0.842 ± 0.04	0.847 ± 0.03	0.842 ± 0.03

## 5 Discussion and future work

This paper has shown that CNNs can provide predictions for aphasia recovery with a balanced accuracy of approximately 0.85. (We do not count the Table 4 results as best performance, since there is the possibility of over-fitting through feature selection, as we do not cross validate.) The best mean accuracy performance came from using 2D Hybrid ROI images that combined a small number of grey matter ROIs with three tabular features (initial severity of aphasia after stroke, left hemisphere lesion size and recovery time). Of these three features, left hemisphere lesion size was least important when damage to key anatomical regions of interest was incorporated. It may seem surprising that the 3D CNNs were outperformed (in terms of mean accuracy) by some of the 2D models. A key issue is likely to be the number of patients in the dataset. The number of voxels/features in an MRI scan massively exceeds the number of patients and this may lead to the curse of dimensionality. It could be that far larger patient numbers are needed to adequately populate the high dimensional feature space. Problems are further exacerbated by the large number of trainable parameters in standard 3D CNNs. The lightweight 3D ResNet in Wolf et al.’s (2022) DAFT implementation might mitigate against the trainable parameters problem, but risks losing some of the predictive power of deeper ResNet models.

2D CNNs trained on the Stitched MRI dataset had similar mean accuracies to the 3D Resnet trained on the 3D MRI scans. The stitched MRI contain less information than the 3D MRI scans, as they only display 64 MRI slices that are downsized to 256 x 256 images. Yet this loss of information appears to be offset by having a smaller feature space and having less trainable parameters.

Table 1′s mean accuracy results point to the Hybrid ROIs and ROIs datasets having greater prognostic information than their respective Stitched MRI datasets; and in the former case, Hybrid-ROIs w/ResNet-18 vs Stitched MRI w/ResNet18, we could show a statistic difference (see appendix A2). As Fig. 2 illustrates, this is the case even when the number of ROIs being displayed is only four, highlighting the benefit feature selection through explainable AI can bring.

Importantly, the goal of the work reported here was to obtain high classification accuracy and look at which tabular data improved the classifications. This was done in the context of assessing the effectiveness of deep learning, applied to MRI stroke data. In particular, in this paper, we are not illuminating the key, but difficult, question of explaining how the classifier has used the features available to it – either those in the MRI scans or the tabular features.

Accordingly, we are not providing an exact description of how different regions enable good performance. This is consistent with prior studies, which have typically not been able to explain the critical combination of damage behind their predictions. Thus, we are looking at the combination of features, not which ones are dominating.

Indeed, incontrovertible feature importance is difficult to determine, since our data features are fundamentally colinear – the smoothness in brain scans and the stereotypicality of brain damage ensure this. So, it is always the case that the ResNet can extract the same information in different ways. Indeed, the information contained in our tabular features are certainly highly correlated with the information contained in the brain scans. The purpose of taking a multimodal approach is to benefit from all available data. Research on what might be the best way of implementing a multimodal approach is in its infancy. The improvement of accuracy obtained by the Hybrid ROI ResNet18 in comparison with the Early Fusion ResNet18 indicate that the idea of incorporating tabular data as image artefacts can be promising in the case of medical applications of CNNs.

Issues of one modality dominating another can arise in multimodal approaches, but this is typically considered in the context of multimodal fusion at later stages of the classification pathway. Our approach, in which all relevant features are incorporated into the same embedding space (by being in the same images), may limit such issues. Additionally, differences in “detectability” of different visual features is already inherent to the basic visual classification problem that CNNs are trying to handle; for example, even when classifying brain scans on their own, there will be particular features in images that are easy for a CNN to detect and others that are considerably harder to detect.

Indeed, our expectations are that the shapes used to represent the tabular data are so much easier for the ResNet to detect than features in the brain scans that shapes will provide the dominant features driving classification, when tabular data is represented as shapes in brain images. This might mean that features in the scans become less important for the classification performance in our (shape-embedded) multimodal approach. Further work is ongoing in this area.

A key difficulty with the application of machine learning in neuroimaging (and more broadly) is the potential for over-fitting to creep in un-noticed (Hosseini et al., 2020). The difficulty is reflected in the bias-variance dilemma (Kohavi & Wolpert, 1996) (and its “twin”: the trade-off between type-I and type-II errors (Lieberman & Cunningham, 2009)), i.e. changes that increase classification accuracy have the potential to hinder generalisation, or in other words, efforts to reduce under-fitting, can increase over-fitting. This is essentially because some of the improvement in classification accuracy is due to finding pattern in noise, rather than in signal. Additionally, this problem is especially serious when datasets are small, which in machine learning terms, ours is. The problem is that, with small data, the effective signal-to-noise ratio is also small. However, we believe that we have been diligent in protecting ourselves against gross overfitting. For example, use of a lock-box, which is only opened once (Hosseini et al.,2020), suggests that our reported accuracies reliably reflect the out-of-sample effectiveness of our learning algorithms, given the data available to us.

There is a subtle issue that if the (out-of-sample) accuracies of multiple learning algorithms are quantified on the *same* lock-box, the choice of the best amongst these will be inflated by this multiple testing. Ideally, one would like to have two lock-boxes, one to determine the best algorithm and a second to determine its true out-of-sample accuracy. However, if you are choosing between a relatively small number of algorithms (we have 10), using a single lock-box is not likely to be a large inflation of accuracy. All this said, replication in a new dataset, preferably by a new research group, is the ultimate test of generalization. We await this assessment.

Notably, in this paper, logistic regression classified surprisingly well (see Table 1, Table 2) given that it only includes one coarse imaging variable: lesion size. For example, “Early Fusion Hybrid w/ResNet-18” has a performance below logistic regression; although, we were able to show that our best model, 2D Hybrid ROIs w/ResNet-18, did perform better than logistic regression in a statistical sense; see appendix A2. Accordingly, in this work, we are not claiming to have established that deep learning substantially outperforms more traditional methods. One possibility is that, as previously noted, even though our data set is big by neuroimaging standards, it remains small by machine learning standards. Accordingly, it may be that subtle spatial patterns of brain damage, can only benefit deep learning with the higher signal-to-noise afforded by larger data sets. This is a question we are actively pursuing. Put in other terms, with our data size, it may be that the curse of dimensionality is limiting our capacity to train the larger (deep learning) models. Additionally, incorporating further symbolic knowledge and explainability may help with this problem, as advocated in neurosymbolic AI (d’Avila Garcez et al., 2002).

In this respect, there appears to be significant potential for increasing the predictive accuracy of CNN models for aphasia recovery. For example, the PLORAS dataset is planned to include an additional 2000 S survivors by 2028. Increasing the dataset size might reduce some of the problems with the curse of dimensionality and the large number of trainable parameters. The larger datasets may also improve the 2D CNNs’ ability to learn complex patterns in the data, reflecting the heterogeneous nature of lesion patterns generating a particular deficit.

There is considerable scope for further developing the hybrid image approach. For example, additional tabular features could be included such as age, sex at birth, handedness and the duration and intensity of treatments. Nonlinear transformations could also be applied to some of the tabular features, with the new values being represented by changes in the corresponding symbols’ intensities or sizes. Symbolic data could also be added to the 3D MRI scans. Finally, hybrid images could be created that combine grey and white matter as well as tabular data.

One reason we are able to obtain relatively high accuracies is due to the pre-training of the ResNet on the ImageNet dataset. For example, using pre-training weights improved the balanced accuracy of the Hybrid ROIs from 0.823 to 0.855. However, this pre-training is not focused on images relevant to the learning problem being considered, i.e. the networks were not trained on brain-scans. Consequently, if a very large dataset of T1-weighted MRI scans (hopefully, of 100 s of thousands) can be identified then it may be possible to provide a pre-training that tunes the convolutional kernels to features more appropriate for classification from the brain-scans available from stroke patients. There are a number of ways in which a teacher signal can be obtained for this pre-training as part of a so-called teacher-student approach (Doersch et al., 2015). For example, the student CNN to be pre-trained could become the encoder in an autoencoder architecture, with the input scans, or parts of them, also serving as teacher pattern (Pathak et al., 2016). Additionally, if suitable cognitive measures are available with the pre-training dataset, then they could be used as the teacher signal. If available, training to classify language abilities should tune the CNN kernels very appropriately for classifying stroke recovery.

Finally, PLORAS is now collecting longitudinal data from 90 aphasic stroke survivors, including both MRI scans and extended tabular data. Changes in the voxel intensities and tabular features may well be prognostically valuable. These changes could be incorporated into hybrid images.

## 6 Limitations

A key limitation of this work is that it has been restricted to research quality MRI scanners. If CNNs are to be clinically employed, then they will need to achieve high levels of accuracy using images from hospital scanners, including CT images. In addition, the images are collected on research scanners at a mean of 46.38 (standard deviation 54.21) months after the stroke. Thus, imaging and CAT scores are collected later than initial severity. Future work needs to classify from clinical imaging collected soon after the stroke.

Additionally, in this paper we have followed other studies in using anatomical atlases (Hope et al., 2013, Hope et al., 2015, Hope et al., 2018). Future studies could use functional parcellation, but there are also limitations with these because they do not correspond to the vascular territories that determine stroke damage.

An important further issue is that our measure of initial severity is relatively crude (see description in second paragraph of subsection 2.1). However, although currently unpublished, within the PLORAS research programme, we have found the measure to be reliable and effective. This is consistent with our findings in this paper. Indeed, in a sense, “the proof is in the pudding”; that is, even if our measure of initial severity is crude, it seems to carry considerable information that classifiers, whether traditional linear approaches (such as logistic regression) or modern non-linear methods (such as deep learning), can use to classify patients. Although, there is certainly a good deal of further work required, such as relating our measure of initial severity to what would typically be considered more objective measures such as, NIHSS or standardised speech and language assessments.

Importantly, spoken picture description performance is measured across a range of values, which are often predicted using regression. In this paper, this scale has been simplified to a binary classification task. This is because the majority of deep learning techniques are targeted at classification, rather than prediction of a continuous dimension, as performed by regression. Support vector machine regression exists (Awad et al., 2015), and is a powerful approach, but it is not a deep learning approach in the sense we are focussed on in this paper.

We could have attempted to extract continuous (regression-like) predictions from a late layer of the ResNet, but such approaches are currently somewhat ad hoc, and may not work well. This is because the neural network has been trained to classify, not predict on a continuous scale, and the layer being predicted from is unlikely to be well tuned for this “secondary” task. Our specific objective for this paper was to provide a state-of-the-art assessment of the effectiveness of modern deep learning when applied to assessing recovery from stroke. This is unlikely to be well served by bolting an ad hoc mechanism onto a deep learning network.

However, it is also interesting to consider the effect on predictive performance that might follow from different ways of encoding the Comprehensive Aphasia test T-scores, with binary classification being an extreme form of such encoding, i.e. into two bins. On the one hand, grouping or binning the scores might obscure the signal if within-bin differences are systematic and predictable. But on the other hand, this grouping might enhance the signal if within-bin differences are driven by measurement noise, or are otherwise not predictable given the predictor variables that we have.

Our investigation of this issue has yielded preliminary results suggesting that in fact, this encoding makes little difference overall, yielding a numerical (non-significant) advantage for classification over regression. In the context of deep learning, this advantage might simply reflect the broader trend that more research effort has been devoted to classification than to regression. However, this investigation is not currently published.

Additionally, some sort of binning seems required, since there is often no easy way to interpret very small T-score differences in ways that matter to patients. In this sense, a shift to more categorical approaches is motivated both by available methodology (deep learning works better for classification), and by clinical need. Naturally, as just discussed, binning into two classes might obscure useful information, but some binning will be required, and the binary one we have used here may be the only one that can be made without a lot of argument, because it comes out of the standardisation of the original task. More detailed binning needs more justification.

This is undoubtedly an important topic for further work, especially since patients and clinicians will want estimates of how severe an impairment would be.

A general limitation of using CNNs is that the complexity of their calculations is beyond human capacities to understand. Yet in a clinical setting it seems essential to be able to understand and explain why particular predictions are being made. This highlights the need for explainable AI methods. There are many explainable AI methods available. This paper has used a bespoke version of CLEAR Image, other methods include Grad-CAM (Selvaraju et al., 2017) and LIME (Ribeiro et al., 2016). Unfortunately, these methods often differ in their putative explanations of a CNN’s classification probabilities, highlighting different regions as being important (Fong et al., 2019, White et al., 2023). A priority for explainable AI methods then, is to show that their explanations are faithful, i.e. they correctly mimic the input–output behaviour of the AI classifier that they are meant to be explaining. CLEAR Image does provide fidelity statistics unlike Grad-CAM or LIME. However, further work is needed to assess the fidelities of explainable AI methods with MRI data and the relevance of the brain regions and counterfactuals highlighted by CLEAR Image.

There are two potential criticisms in the scope of the paper’s experiments that were judged to be of low risk. The first is that there may be a different CNN architecture (e.g. EfficientNet) or a Transformer Network that would have produced better results. However, we are unaware of any papers that indicate that a different architecture would be expected to generate substantially improved results compared to using ResNet models in this area of application. It could also be argued that the project should have used data augmentation to increase the size of the training datasets. However, we are training CNNs to discriminate between small changes in lesion sizes and locations on spatially normalised images. The usual data augmentation transformations of rotation, blurring and translation would have distorted the subtle patterns that the CNN needed to learn (see Wang et al. (2023) for a similar argument when using CNNs to detect patterns in MRI scans of Alzheimer patients).

## 7 Conclusions

Predicting recovery from post-stroke aphasia could enable targeted therapy. This paper has provided an evaluation of the effectiveness of deep learning for predicting the class of a patient’s spoken picture description score (i.e. aphasic/non-aphasic). We have provided evidence that deep learning with ResNets, multimodal data and feature selection using explainable AI can achieve high levels of predictive accuracy. Importantly, though, our accuracies will have benefitted from the careful balancing that we performed of our five groups (on recovery time, initial severity, left lesion size and spoken picture description scores), which, for example, is likely to have meant that our training will have been appropriate for the distributional properties of our lock-box. Additionally, if deep learning methods are to be clinically employed, they will need to achieve high levels of accuracy using images from hospital scanners. There appears to be significant potential for achieving this, for example by increasing dataset size, developing the hybrid image approach as a neurosymbolic system, better pre-training weights and using longitudinal data. Our findings may also be relevant to other neuroscience fields that wish to combine image data and tabular data. In cases where a dataset is small in machine learning terms, our novel approach of training a CNN on images that combine ROIs with symbolic representations of tabular data may be fruitful.

## CRediT authorship contribution statement

**Adam White:** Writing – original draft, Software, Methodology, Formal analysis, Conceptualization. **Margarita Saranti:** Writing – review & editing, Software, Methodology, Conceptualization. **Artur d’Avila Garcez:** Writing – review & editing, Validation, Methodology. **Thomas M.H. Hope:** Writing – review & editing, Methodology, Conceptualization. **Cathy J. Price:** Writing – review & editing, Supervision, Resources, Funding acquisition, Data curation, Conceptualization. **Howard Bowman:** Writing – review & editing, Supervision, Software, Methodology, Conceptualization.

## Figures and Tables

**Fig. A1:**
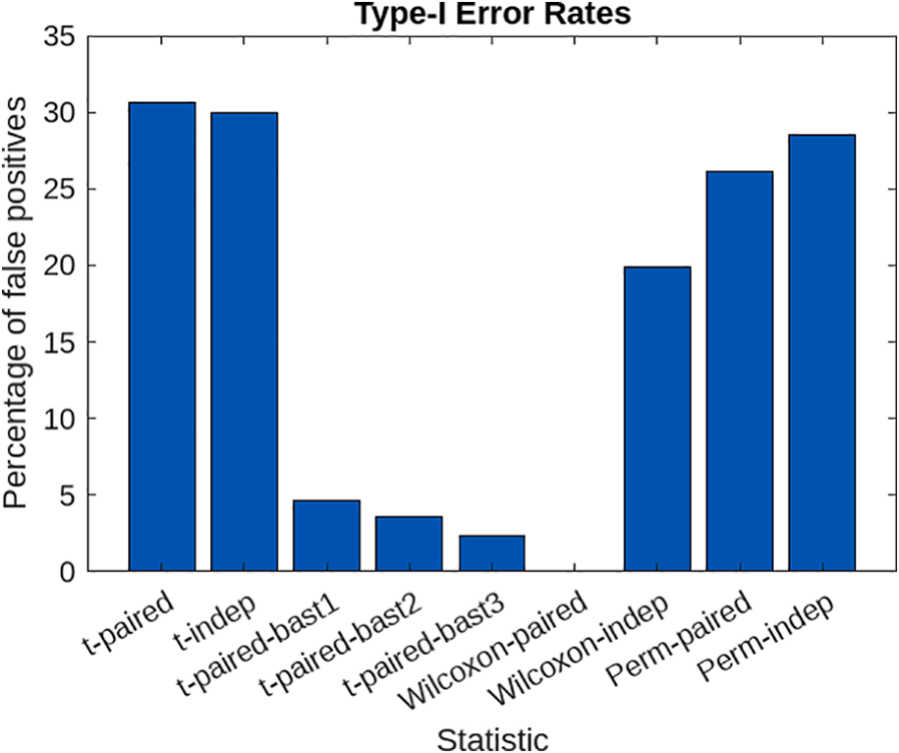
Results of false positive test. All tests were two-tailed. Mostly paired tests are presented, but we include independent tests for standard t, Wilcoxon and permutation. This is because the paired Wilcoxon fails as a result of insufficient orderings of four items (in fact, permutation-paired faces a similar shortage of orderings, but manages to provide p-values by virtue of the variably introduced by the Monte-Carlo resampling). We wanted to make clear that the problem being considered is not due to loss of normality, i.e. Wilcoxon-independent, as well as the permutation procedures (none of which make normality assumptions), also exhibit very substantial inflation of the false positive rate. t-paired-bast1, t-paired-bast2 and t-paired-bast3 are the new method with corrected degrees of freedom. These are scaled down to three different levels downScale1 = 0.45, downScale2 = 0. 435 and downScale3 = 0.4. t-paired-bast1 has the closest type-I error rate to alpha; it is 4.65 %, i.e. just below 5 % = alpha × 100.

**Table A2.1:** Statistical inference on across folds balanced accuracies of Hybrid ROIs w/ResNet-18 against all other models (with each row this contrast for one model). Table 1 in main body of paper shows the descriptive statistics for the same models. Unlike in Table 1, here, models are ordered from largest (adjusted) t-value to smallest, which also sorts p-values smallest to largest. Mean accuracies and standard deviations are as presented in Table 1. t-stat (adj) is the t-statistic introduced in appendix A1, with degrees of freedom adjusted using the scaling factor 0.45. p-value is the corresponding p-value, calculated from t-stat (adj), with adjusted degrees of freedom. BH threshold is the False Discovery Rate (FDR) adjustment of a 0.05 statistical threshold, with nine comparisons, using the Benjamini–Hochberg procedure (Benjamini and Hochberg, 1995). BH p-adj is the adjusted p-value implied by the Benjamini–Hochberg procedure; accordingly, these p-values can be considered relative to a 0.05 threshold.

Hybrid ROIs w/ResNet-18 vs < Model>	Mean accuracy	Standard deviation	t-stat (adj)	p-value	BH threshold	BH p-adj
Hybrid Stitched MRI w/Lightweight CNN	0.819	0.001	21.357	0.011	0.006	0.102
Stitched MRI w/Lightweight CNN	0.801	0.002	20.576	0.012	0.011	0.054
Stitched MRI w/ResNet-18	0.807	0.003	17.986	0.014	0.017	0.043
Logistic regression	0.810	0.003	14.336	0.019	0.022	0.044
Early Fusion Hybrid w/ResNet-18	0.800	0.004	9.171	0.035	0.028	0.063
DAFT	0.814	0.003	9.033	0.036	0.033	0.054
MRI Scans w/ResNet3D	0.805	0.004	7.706	0.044	0.039	0.057
ROIs w/ResNet-18	0.811	0.005	5.434	0.070	0.044	0.079
Hybrid Stitched MRI w/ResNet-18	0.829	0.004	4.062	0.103	0.050	0.103
**Hybrid ROIs w/ResNet-18**	***0.854***	***0.001***				

## Data Availability

The data will be made available upon reasonable request, material transfer agreements, and time involved in data preparation. The code is available at https://github.com/ClearExplanationsAI/CLEAR-MRI.

## Appendix A1 : Verifying statistical procedure

**t-tests**

Statistical inference to demonstrate that one machine learning algorithm is superior to another has its challenges (Dietterich, 1998). Firstly, as with statistical inference in many other contexts, one would really like to test significance where the unit of statistical inference is the participant. However, measures of performance of machine learning algorithms are (typically) only obtained across the entire cohort of participants. One could employ a resampling procedure to obtain surrogate datasets. However, this would require the machine learning algorithms to be trained over each one of these surrogate datasets, requiring potentially many hundreds or even thousands of re-trainings of the algorithms, which is infeasible.

Accordingly, one typically performs inference across the folds arising from a cross validation procedure. However, this has its own difficulties, because (apart from in the split-half case) folds are not independent samples. For example, in fivefold cross validation, the training sets in any two folds share 3/4 of their data, i.e. three of the five disjoint partitions of the data. This breaks the IID (identically independently distributed) assumption of standard statistical tests, and will increase type 1 error rates; see below.

(Dietterich, 1998) is a key paper, which demonstrates this inflation with standard procedures and provides simulation evidence that their 5×2 cross validation test does not inflate false positive rates. Notably, this procedure removes the $\sqrt{n}$ term from the classical *t*-test. This term scales the remainder of the t- formula (which is just a standardised effect size) according to the square root of the degrees of freedom (plus one).

This, we believe, indicates the direction for responding to the non-independence of folds. Essentially, because of the correlation between folds, one does not have as many degrees of freedom as the standard *t*-test formula suggests. Accordingly, a correction needs to be applied to the degrees of freedom. Although applied in different contexts, i.e. ANOVAs that do not correspond to t-tests, corrections for loss of sphericity, such as Greenhouse Geiser corrections to the degrees of freedom, address a similar point.

Accordingly, we have simulated our cross-validation procedure under the null. The simulations were set-up as follows:

1) Noise data of 18 dimensions was randomly sampled from independent gaussians, i.e. noise is i.i.d (independently identically distributed). 960 observations were generated (i.e. close to the sample size in this paper, but divisible in the way needed for these simulations).

2) Half of these observations were given class label 1; the other half class label 2.

3) The simulations contained “signal”, since for class 1, a random pattern was generated across dimensions, and then included unchanged in every class 1 observation. The same was done for class 2. This consistent pattern was overlaid with the noise from point 1).

4) In order to ensure the null was true (i.e. the two classifiers being compared were not different) we used the same algorithm (linear discriminant analysis) for both the classifiers. Statistical differences between the two classifiers were obtained through feature selection: with a proportion of the input dimensions ignored by each classifier. This proportion was the same for both classifiers, but the units ignored were non-overlapping.

5) The null hypothesis should be true in all simulations; i.e. since everything was randomly sampled, the algorithms were the same for both classifiers and the proportions left out were the same size.

6) K fold cross validation was implemented, with simulations reported here employing 4 folds, the situation applied in this paper.

7) A lock-box, of the size of a single fold, with the properties of the rest of the data was generated. The final test accuracy of each fold was calculated on this lock-box.

8) We simulated the null 800 times in order to assess the type-I error rate, which given an alpha level of 0.05 should yield close to 40 significant p-values.

The use of a lock-box in our analysis procedure, increases the correlation between folds, making the loss of iid more severe.

The standard t-statistic for a paired test can be expressed as follows:

$$t=\frac{\mu_{\mathrm{diff}}.\sqrt{\mathrm{df}+1}}{\sqrt{\frac{\sum_{i=1}^{N}{\left({\mathrm{diff}}_{i}-\mu_{\mathrm{diff}}\right)}^{2}}{N-1}}}$$

where, N is the sample size; df=N-1 is the degrees of freedom; ${\mathrm{diff}}_{i}=x_{i}-y_{i}$ is the difference between the two conditions (x and y) for the i th sample; and $\mu_{\mathrm{diff}}$ is the mean of these differences across all samples. This statistic is distributed as t with df degrees of freedom.

The df-corrected t-statistic that we use scales df in this equation. Thus,

$$\mathrm{df}={\mathrm{df}}_{\mathrm{corrected}}=s.\left(N-1\right)$$

which is distributed as t with ${\mathrm{df}}_{\mathrm{corrected}}$ degrees of freedom. s has to be between $\frac{1}{\left(N-1\right)}$ and one, i.e. corrected degrees of freedom cannot be less than 1 or bigger than the original dof. Then, the p-value is inferred as one minus the t-distributions’s cumulative distribution function applied to t with ${\mathrm{df}}_{\mathrm{corrected}}$ degrees of freedom.

Our main findings are shown in figure A1.

So, all traditional methods (paired and independent t; independent Wilcoxon; and paired and independent permutation) inflate the false-positive rate very considerably (paired Wilcoxon would do as well, but with just four, there are insufficient samples to calculate it).

The degrees of freedom correction we are proposing, returned the paired *t*-test to a non-inflating regime. That is, if we scale the degrees of freedom by 0.45, we obtain a type-I error rate close to alpha, i.e. 4.65 %. The scalings of 0.435 and 0.4 indicate the sensitivity to changes in this scaling factor.

On the basis of these simulation results, we employ a scaling of 0.45 in the statistical inference proper we perform in appendix A2 Thus, s in our degrees of freedom correction becomes 0.45, i.e.

$$\mathrm{df}={\mathrm{df}}_{\mathrm{corrected}}=s.\left(N-1\right)=0.45.\left(N-1\right)$$

and the t-formula is unchanged in all other respects.

**Confidence intervals**

We can also calculate confidence intervals that are adjusted for the reduced degrees of freedom arising from folding. That is, we can calculate the standard error of the mean as,

$${\mathrm{sem}}_{x}=\frac{{\mathrm{std}}_{x}}{\sqrt{\mathrm{df}+1}}=\frac{\sqrt{\frac{\sum_{i=1}^{N}{\left(x_{i}-\mu_{x}\right)}^{2}}{N-1}}}{\sqrt{\mathrm{df}+1}}$$

where $x_{i}$ is the accuracy of the i th fold; $\mu_{x}$ is the mean across fold accuracies; N is the sample size; and for us,

$$\mathrm{df}={\mathrm{df}}_{\mathrm{corrected}}=s.\left(N-1\right)$$

where s is the down scaling of the degrees of freedom. We also need the critical t-value for a probability of 0.025; which can be obtained as,

$$\mathrm{ts}=tinv(0.025,{\mathrm{df}}_{\mathrm{corrected}})$$

where tinv is the inverse of the t statistic’s cumulative distribution function.

Then, our confidence intervals are:

$$\mathrm{CI}=\;\mu_{x}\mp \;ts.{\mathrm{sem}}_{x}$$

## Appendix A2 : Statistical results

We now apply the correction identified in appendix A1 to the across fold accuracies from which descriptive statistics are presented in Table 1. We especially focus on the “all patients” balanced accuracy, which is a particularly informative measure.

In order to statistically compare against it, we re-ran logistic regression classification, but this time with folding. Table 1, Table 2 show logistic regression’s classification performance when it is run over the four splits of the data that are available once the lock-box split is removed. Thus, there was no division of the four into training and validation, logistic regression was simply fit to all four splits put together. This made logical sense, since these are what are available because, with logistic regression, there is no necessity to use a validation set to determine when learning should stop. However, this strategy is not sufficient to enable a statistical comparison of logistic regression to the other models, since that is performed across folds.

Accordingly, we refit the logistic regression but this time we fit four times, once on each of the ways three splits can be taken from the four left after the lock-box is removed. A consequence of this is that in each of these four foldings, one split of the data (which would have been the validation set) is unused. This then gave us a logistic regression accuracy for each fold, which had a mean of 0.810 and a confidence interval of 0.034. It is this logistic regression fitting that we statistically compare to the other models here.

The statistical inferences arising from this correction are presented in Table A2.1. The t-stat (adj) and p-value columns present the results of applying the degrees of freedom adjustment of appendix A1. We follow this with a False Discovery Rate correction (Benjamini and Hochberg, 1995) for multiple comparisons, in the columns BH threshold and BH p-adj.

This enables us to consider which of our models have accuracies that our best model (Hybrid ROIs w/ResNet-18, last row) is better than. (Formulating inference in this way is convenient, since it means that all our tests will be positively dependent, which is a pre-requisite for applying False Discovery Rate multiple comparisons correction (Benjamini and Yekutieli, 2001) in the way we have.) We highlight the four comparisons that are significant according to the FDR correction. Importantly, even though the models of the first two rows do not cross their individual (adjusted) statistical threshold, as they are followed by models that do, according to the FDR logic, they are counted as significant.

Perhaps most notably, logistic regression is one of these four. Thus, we have obtained evidence to reject the null hypothesis that our best model (Hybrid ROIs w/ResNet-18) has the same accuracy as our baseline model, logistic regression.

## References

[b0005] Altman N., Krzywinski M. (2018). The curse (s) of dimensionality. Nat. Methods.

[b0010] Ashburner J., Friston K.J. (2005). Unified segmentation. Neuroimage.

[b0015] Awad, M., Khanna, R., Awad, M., & Khanna, R. (2015). Support vector regression. Efficient learning machines: Theories, concepts, and applications for engineers and system designers, 67-80.

[b0020] Benghanem S., Rosso C., Arbizu C., Moulton E., Dormont D., Leger A., Samson Y. (2019). Aphasia outcome: the interactions between initial severity, lesion size and location. J. Neurol..

[bib216] Benjamini Y., Hochberg Y. (1995). Controlling the false discovery rate: a practical and powerful approach to multiple testing. J. Roy. Stat. Soc. Ser. B (Met.).

[bib217] Benjamini Y., Yekutieli D. (2001). The control of the false discovery rate in multiple testing under dependency. Ann. Stat..

[b0025] Bonkhoff A.K., Hope T., Bzdok D., Guggisberg A.G., Hawe R.L., Dukelow S.P., Bowman H. (2020). Bringing proportional recovery into proportion: Bayesian modelling of post-stroke motor impairment. Brain.

[b0030] Bowman H., Bonkhoff A., Hope T., Grefkes C., Price C. (2021). Inflated estimates of proportional recovery from stroke: the dangers of mathematical coupling and compression to ceiling. Stroke.

[b0035] Chauhan S., Vig L., De Filippo De Grazia M., Corbetta M., Ahmad S., Zorzi M. (2019). A comparison of shallow and deep learning methods for predicting cognitive performance of stroke patients from MRI lesion images. Front. Neuroinf..

[bib211] Chen, S., Ma, K., Zheng, Y., 2019. Med3d: Transfer learning for 3d medical image analysis. *arXiv preprint arXiv:1904.00625*.

[b0040] Crinion J., Ashburner J., Leff A., Brett M., Price C., Friston K. (2007). Spatial normalization of lesioned brains: performance evaluation and impact on fMRI analyses. Neuroimage.

[b0045] d’Avila Garcez A., Broda K.B., Gabbay D. (2002).

[b0050] Deichmann R., Schwarzbauer C., Turner R. (2004). Optimisation of the 3D MDEFT sequence for anatomical brain imaging: technical implications at 1.5 and 3 T. Neuroimage.

[bib212] Dietterich T.G. (1998). Approximate statistical tests for comparing supervised classification learning algorithms. Neural Comput..

[b0055] Doersch C., Gupta A., Efros A.A. (2015). Proceedings of the IEEE International Conference on Computer Vision.

[b0060] Esmaeilzadeh S., Belivanis D.I., Pohl K.M., Adeli E. (2018). Machine learning in medical imaging: 9th International workshop, MLMI 2018, held in conjunction with MICCAI 2018, Granada, Spain, September 16, 2018, Proceedings 9.

[bib214] Fong R., Patrick M., Vedaldi A. (2019). Proceedings of the IEEE/CVF international conference on computer vision.

[bib209] Guo C., Pleiss G., Sun Y., Weinberger K.Q. (2017, July). International conference on machine learning.

[b0065] Hope T.M., Seghier M.L., Leff A.P., Price C.J. (2013). Predicting outcome and recovery after stroke with lesions extracted from MRI images. NeuroImage: Clinical.

[b0070] Hope T.M., Parker Jones Ō., Grogan A., Crinion J., Rae J., Ruffle L., Green D.W. (2015). Comparing language outcomes in monolingual and bilingual stroke patients. Brain.

[b0075] Hope T.M., Leff A.P., Price C.J. (2018). Predicting language outcomes after stroke: Is structural disconnection a useful predictor?. NeuroImage: Clinical.

[b0080] Hope T.M., Friston K., Price C.J., Leff A.P., Rotshtein P., Bowman H. (2019). Recovery after stroke: not so proportional after all?. Brain.

[b0085] Hosseini M., Powell M., Collins J., Callahan-Flintoft C., Jones W., Bowman H., Wyble B. (2020). I tried a bunch of things: The dangers of unexpected overfitting in classification of brain data. Neurosci. Biobehav. Rev..

[b0090] Huang S.C., Pareek A., Seyyedi S., Banerjee I., Lungren M.P. (2020). Fusion of medical imaging and electronic health records using deep learning: a systematic review and implementation guidelines. npj Digital Med..

[b0095] Johnson L., Nemati S., Bonilha L., Rorden C., Busby N., Basilakos A., Fridriksson J. (2022). Predictors beyond the lesion: health and demographic factors associated with aphasia severity. Cortex.

[b0100] Kohavi R., Wolpert D.H. (1996). Bias plus variance decomposition for zero-one loss functions. ICML.

[b0105] Lazar R.M., Minzer B., Antoniello D., Festa J.R., Krakauer J.W., Marshall R.S. (2010). Improvement in aphasia scores after stroke is well predicted by initial severity. Stroke.

[b0110] Lieberman M.D., Cunningham W.A. (2009). Type I and Type II error concerns in fMRI research: re-balancing the scale. Soc. Cogn. Affect. Neurosci..

[b0115] Liu M., Zhang J., Adeli E., Shen D. (2018). Joint classification and regression via deep multi-task multi-channel learning for Alzheimer's disease diagnosis. IEEE Trans. Biomed. Eng..

[bib213] Lorca-Puls D.L., Gajardo-Vidal A., White J., Seghier M.L., Leff A.P., Green D.W., Crinion J.T. (2018). The impact of sample size on the reproducibility of voxel-based lesion-deficit mappings. *Neuropsychologia*.

[b0120] Menahemi-Falkov M., Breitenstein C., Pierce J.E., Hill A.J., O'Halloran R., Rose M.L. (2022). A systematic review of maintenance following intensive therapy programs in chronic post-stroke aphasia: importance of individual response analysis. Disabil. Rehabil..

[b0125] Pathak D., Krahenbuhl P., Donahue J., Darrell T., Efros A.A. (2016). Proceedings of the IEEE Conference on Computer Vision and Pattern Recognition.

[b0130] Penny W.D., Friston K.J., Ashburner J.T., Kiebel S.J., Nichols T.E. (2011). Statistical Parametric Mapping: The Analysis Of Functional Brain Images.

[b0135] Pierce J.E., OHalloran R., Togher L., Nickels L., Copland D., Godecke E., Meinzer M., Rai T., Cadilhac D.A., Kim J., Hurley M., Foster A., Carragher M., Wilcox C., Steel G.R., Rose M.L. (2023). Acceptability, feasibility and preliminary efficacy of low-moderate intensity Constraint Induced Aphasia Therapy and Multi-Modality Aphasia Therapy in chronic aphasia after stroke. Top Stroke Rehab..

[bib210] Raghu M., Zhang C., Kleinberg J., Bengio S. (2019). Advances in neural information processing systems.

[b0145] Ribeiro M.T., Singh S., Guestrin C. (2016). Proceedings of the 22nd ACM SIGKDD international conference on knowledge discovery and data mining.

[b0150] Roberts S., Bruce R.M., Lim L., Woodgate H., Ledingham K., Anderson S., Price C.J. (2022). Better long-term speech outcomes in stroke survivors who received early clinical speech and language therapy: What’s driving recovery?. Neuropsychol. Rehabil..

[b0155] Roohani, Y. H., Sajid, N., Madhyastha, P., Price, C. J., & Hope, T. M. (2018). Predicting language recovery after stroke with convolutional networks on stitched MRI. *arXiv preprint arXiv:1811.10520*.

[bib207] Seghier M.L., Patel E., Prejawa S., Ramsden S., Selmer A., Lim L., Price C.J. (2016). The PLORAS database: a data repository for predicting language outcome and recovery after stroke. Neuroimage.

[b0160] Seghier M.L., Price C.J. (2023). Interpreting and validating complexity and causality in lesion-symptom prognoses. Brain Commun..

[b0165] Seghier M.L., Ramlackhansingh A., Crinion J., Leff A.P., Price C.J. (2008). Lesion identification using unified segmentation-normalisation models and fuzzy clustering. Neuroimage.

[b0170] Selvaraju R.R., Cogswell M., Das A., Vedantam R., Parikh D., Batra D. (2017). Proceedings of the IEEE International Conference on Computer Vision.

[bib208] Swinburn K., Porter G., Howard D. (2004). Comprehensive aphasia test. [Database record]. APA PsycTests.

[b0175] Thye M., Mirman D. (2018). Relative contributions of lesion location and lesion size to predictions of varied language deficits in post-stroke aphasia. NeuroImage: Clinical.

[b0180] Tzourio-Mazoyer N. (2002). Automated anatomical labelling of activations in SPM using a macroscopic anatomical parcellation of the MNI MRI single-subject brain. Neuroimage.

[bib215] Wang D., Honnorat N., Fox P.T., Ritter K., Eickhoff S.B., Seshadri S., Alzheimer’s Disease Neuroimaging Initiative (2023). Deep neural network heatmaps capture Alzheimer’s disease patterns reported in a large meta-analysis of neuroimaging studies. NeuroImage.

[bib206] White, A., Garcez, A.D.A., 2021. Counterfactual instances explain little. *arXiv preprint arXiv:2109.09809*.

[b0190] White A., Ngan K.H., Phelan J., Ryan K., Afgeh S.S., Reyes-Aldasoro C.C., Garcez A.D.A. (2023). Contrastive counterfactual visual explanations with overdetermination. Mach. Learn..

[b0195] Wolf T.N., Pölsterl S., Wachinger C., Alzheimer’s Disease Neuroimaging Initiative (2022). DAFT: a universal module to interweave tabular data and 3D images in CNNs. Neuroimage.

[b0200] Yu H., Yang L.T., Zhang Q., Armstrong D., Deen M.J. (2021). Convolutional neural networks for medical image analysis: state-of-the-art, comparisons, improvement and perspectives. Neurocomputing.

[b0205] Zhao Y., Cox C.R., Lambon Ralph M.A., Halai A.D. (2023). Using in vivo functional and structural connectivity to predict chronic stroke aphasia deficits. Brain.

